# Stand Age–Associated Rhizosphere Bacterial Succession in the Desert Shrub *Haloxylon ammodendron*

**DOI:** 10.3390/microorganisms14051087

**Published:** 2026-05-11

**Authors:** Zhen Zhao, Weikang Dong, Zhibin Zhou, Jinglong Fan

**Affiliations:** 1 National Engineering Technology Research Center for Desert-Oasis Ecological Construction Technology, Xinjiang Institute of Ecology and Geography, Chinese Academy of Sciences, Urumqi 830011, China; zhaozhen23@mails.ucas.ac.cn (Z.Z.); 18453311731@163.com (W.D.); zhouzb2008@yahoo.com.cn (Z.Z.); 2 University of Chinese Academy of Sciences, Beijing 100049, China; 3 Xinjiang Field Scientific Observation and Research Station of the Taklimakan Desert Ecosystem, Qiemo 841900, China

**Keywords:** *Haloxylon ammodendron*, rhizosphere microbiome, stand age, functional prediction, bacterial succession, community assembly, arid ecosystems

## Abstract

*Haloxylon ammodendron* is a keystone shrub widely used for ecological restoration in arid regions of Northwest China. However, how rhizosphere bacterial communities reorganize across stand ages remains poorly understood. Rhizosphere soils were collected from one-, three-, and six-year-old stands using full-length 16S rRNA sequencing. Although alpha diversity remained relatively stable, beta diversity revealed pronounced community turnover. The dominant phyla were conserved across stands, whereas genus- and species-level composition shifted systematically along the age gradient. Younger stands were enriched in stress-tolerant and early colonizing taxa, intermediate stands showed increased representation of plant-associated and nitrogen-cycling bacteria, and older stands harbored taxa associated with complex carbon turnover and stress adaptation. Network analysis suggested modular co-occurrence patterns across stand ages and PICRUSt2-based functional inference indicated a conserved core metabolic repertoire accompanied by gradual ecological differentiation in pathways related to resource utilization and environmental response. Together, these findings suggest a stand age–associated pattern of rhizosphere bacterial succession and provide insight into microbial community differentiation in a desert shrub system.

## 1. Introduction

In desert ecosystems, plant–microbe–soil interactions play central roles in sustaining ecosystem functions and driving vegetation succession [1,2,3,4]. Soils harbor one of the most diverse and heterogeneous microbial ecosystems on Earth [5,6,7]. The rhizosphere constitutes a key microsite where roots acquire water and nutrients and interact closely with the physical, chemical, and biological components of the soil [8,9,10,11]. In arid lands, microorganisms are major drivers of soil ecological processes, and rhizosphere microbial diversity, composition, and functions often shift with plant species, host developmental stage, and soil conditions [12,13,14,15]. Understanding how plant developmental stages are associated with rhizosphere microbiome variation is essential for interpreting microbial assembly processes in arid ecosystems.

*Haloxylon ammodendron* (*H. ammodendron*), a typical woody desert plant, is highly drought- and salt-tolerant and plays a pivotal role in ecological restoration and sand stabilization in Northwest China [16,17,18]. Rhizosphere microbial communities are associated with nutrient cycling, hormonal signaling, and stress resistance, potentially influencing host growth and environmental adaptation. *H. ammodendron* also serves as a principal host for the parasitic medicinal plant *Cistanche deserticola*, and the host–microbiome context may contribute to host suitability [19,20,21]. Host root exudates may shape the rhizosphere microbiome—e.g., by enriching functional groups such as nitrogen fixers, phosphate solubilizers, and indole-3-acetic-acid (IAA) producers—which could in turn be linked to parasitism establishment and colonization efficiency [22,23,24,25,26]. Therefore, elucidating rhizosphere micro-ecological dynamics across host stand ages may provide an integrative view of the “host age–rhizosphere microbiome–parasitism potential” continuum and may provide a conceptual framework for understanding host age–microbiome interactions [27,28,29].

Over longer timescales, increasing stand age may also be accompanied by changes in soil microbial diversity and the accumulation of pathogenic taxa [30], potentially related to stand decline and root diseases, suggesting that the relationships between stand age [31,32,33], microbiome, and host health can be complex and potentially nonlinear [34]. However, systematic evidence of bacterial functional succession across the young-to-mid-age stages of *H. ammodendron* and its putative implications for host suitability remains limited [35].

Here, we hypothesized that under broadly comparable edaphic backgrounds, increasing stand age would be associated with an ordered transition of the rhizosphere bacterial community from a “salt-tolerant colonizer” assemblage toward a community with enhanced plant-growth promotion and nitrogen cycling, and further toward one characterized by strengthened degradation of complex substrates and stress resistance [36,37,38,39]. Such stage-wise functional shifts may alter rhizosphere nutrients and signaling environments, and could be associated with host suitability for C. deserticola. To test this hypothesis, we combined PacBio CCS full-length 16S rRNA sequencing with community statistics, co-occurrence network inference, and PICRUSt2 functional prediction to characterize age-associated rhizosphere bacterial dynamics and to discuss their potential ecological implications [40,41,42]. Therefore, the objective of this study was to characterize stand age–associated changes in rhizosphere bacterial community structure, co-occurrence patterns, and predicted functional profiles in *H. ammodendron* under arid desert conditions.

## 2. Materials and Methods

### 2.1. Sampling and Soil Physicochemical Measurements

**Figure 1:**
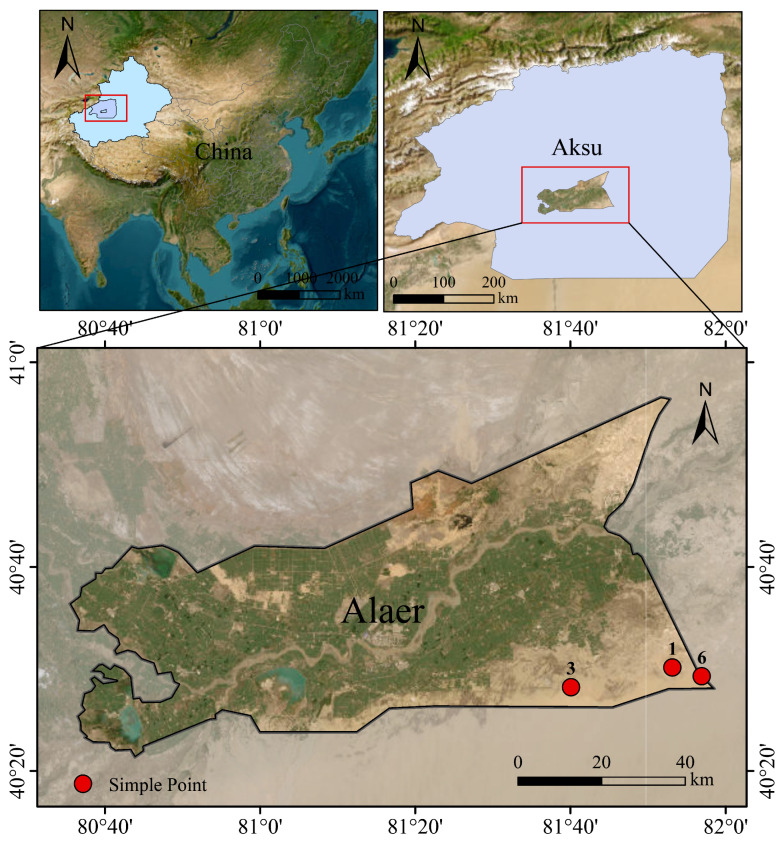
Map of the study area and sampling sites. Numbers 1, 3, and 6 indicate the 1-, 3-, and 6-year-old *Haloxylon ammodendron* stands, respectively. This map was produced based on the standard map with approval number GS(2024)0650, downloaded from the website of the National Geomatics Center of China. The boundaries of the base map were not modified.

Rhizosphere soil was collected from *H. ammodendron* stands of three ages (one-year: AO; three-year: AT; six-year: AS, AO_1–AO_3, AT_1–AT_3, and AS_1–AS_3 represent three biological replicates for each stand-age group.) at the desert margin of the Taklamakan Desert in the Aral region of Xinjiang, China (Figure 1). Sampling was conducted in April 2025. The coordinates of the three stands were: AO (81.88599933° E, 40.50240387° N), AT (81.66818189° E, 40.47023541° N), and AS (81.94893676° E, 40.48808621° N). At each stand age, three healthy plants were randomly selected as biological replicates (n = 9). Soil blocks containing intact roots were excavated; loosely attached bulk soil was gently shaken off; tightly adhering rhizosphere soil was brushed from root surfaces with sterile brushes into sterile centrifuge tubes, immediately snap-frozen in liquid nitrogen in the field, transported on dry ice, and stored at −80 °C until DNA extraction.

In parallel, subsamples were air-dried and passed through a 2-mm sieve for soil physicochemical analyses, including pH, soil organic matter (SOM), total nitrogen (TN), total phosphorus (TP), total potassium (TK), alkali-hydrolyzable nitrogen (AN), available phosphorus (AP), and available potassium (AK). The analytical methods used are listed in Table 1.

### 2.2. DNA Extraction and PacBio Sequencing

Total DNA was extracted from the rhizosphere soils using a DNeasy PowerSoil Kit (QIAGEN, Hilden, Germany). The full-length 16S rRNA gene was amplified using universal primers with sample-specific barcodes. Amplicons were verified by agarose gel electrophoresis, purified, and quantified using AMPure PB beads, pooled in equimolar amounts, and used to construct SMRTbell libraries. Sequencing was performed using a PacBio Sequel II platform (Pacific Biosciences, Menlo Park, CA, USA) to generate high-fidelity circular consensus sequencing (HiFi/CCS) reads. Raw data were de-multiplexed, adapters/primers were removed using SMRT Link, reads were quality- and length-filtered, and chimeric sequences were removed prior to downstream analyses.

### 2.3. Bioinformatics and Statistical Analyses

Operational taxonomic units (OTUs) were generated by clustering sequences with 97% similarity. Although ASV-based approaches can provide higher sequence resolution, OTU clustering was adopted in this study to maintain comparability with previous rhizosphere microbiome studies and to support community-level ecological and network analyses. Therefore, the OTU-based framework was considered appropriate for characterizing broad stand age–associated patterns in the present dataset. Extremely low-abundance OTUs were removed prior to analysis. All samples were rarefied to an equal sequencing depth. Sequences were clustered into OTUs at 97% similarity to maintain comparability with previous rhizosphere studies and to support downstream ecological and network analyses. Alpha diversity indices (Sobs, Chao1, ACE, Shannon, Simpson, and Coverage) were calculated and differences among age groups (AO/AT/AS) were evaluated using the Kruskal–Wallis test. Beta diversity was assessed using Bray–Curtis distances based on OTU relative abundances and visualized using principal coordinate analysis (PCoA) and non-metric multidimensional scaling (NMDS; stress reported). The inter- and intra-group distances are summarized using boxplots. Community composition was summarized at the phylum, genus, and species levels and visualized using stacked bar plots and a z-score-standardized heatmap with hierarchical clustering. Shared and unique OTUs were depicted using Venn and UpSet plots, and group–phylum contributions were visualized using a chord diagram. Differentially abundant taxa were identified using LEfSe (alpha = 0.05; LDA score > 2).

Co-occurrence networks were constructed from OTU relative abundances using Spearman correlations. Low-abundance OTUs were filtered prior to network construction, and correlation significance was assessed with multiple-testing correction. Significant associations were retained to build an undirected network in which positive and negative correlations were interpreted as potential co-occurrence and antagonism, respectively. Network topological properties (e.g., degree, clustering coefficient, and modularity) were calculated to identify hub OTUs and major modules. Functional potential was inferred using PICRUSt2 by mapping representative sequences to KEGG Orthologs (KOs), Enzyme Commission (EC) numbers, KEGG pathways/modules, MetaCyc pathways, and COG categories. The results were normalized to relative abundances and visualized as heatmaps at multiple functional levels [43].

## 3. Results

### 3.1. Rhizosphere Bacterial Diversity and Overall Structure

#### 3.1.1. Alpha Diversity

**Figure 2:**
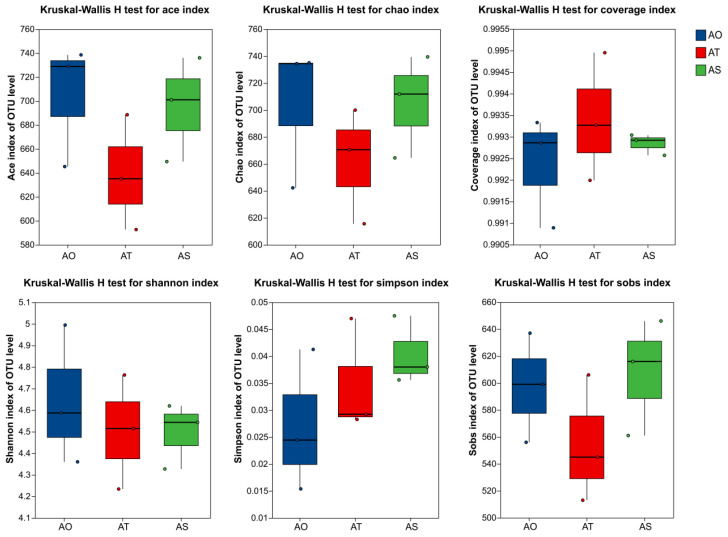
Comparison of alpha diversity indices of rhizosphere bacteria across *H. ammodendron* stand ages.

The coverage values exceeded 99.09% across all samples, indicating that the sequencing depth captured most of the bacterial diversity in the rhizosphere soils (Table 2). The alpha diversity indices did not differ significantly among the stand ages (Figure 2). Chao1 richness ranged from 615.58 to 739.43, Sobs richness from 513 to 646, Shannon diversity from 4.234 to 4.994, Simpson index from 0.015 to 0.048, and ACE from 592.72 to 738.53. Overall, bacterial alpha diversity was high for all three stand ages, suggesting that stand age had a limited effect on alpha diversity at the current sample size and sequencing depth. This pattern is consistent with the possibility that stand age effects are primarily expressed through changes in key taxa and community structure rather than simple richness gains or losses.

#### 3.1.2. Beta Diversity and Clustering

**Figure 3:**
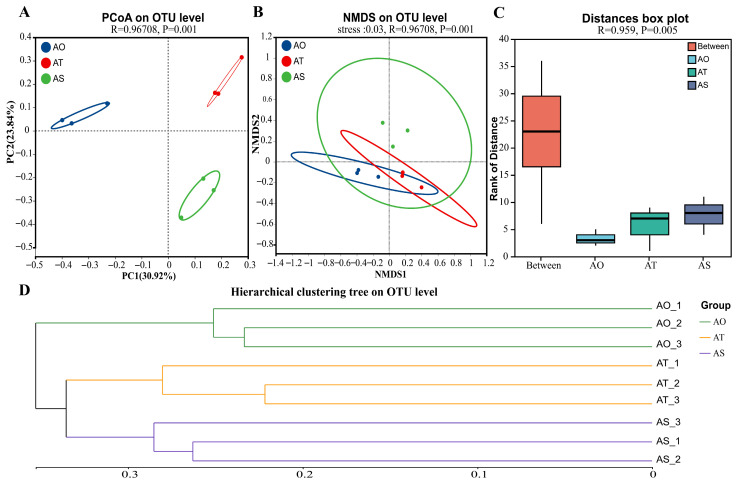
Beta-diversity patterns of rhizosphere bacterial communities across stand ages at the OTU level. (**A**) Principal coordinates analysis (PCoA) based on Bray–Curtis dissimilarities showing separation among AO, AT, and AS groups. (**B**) Non-metric multidimensional scaling (NMDS) analysis based on Bray–Curtis dissimilarities. (**C**) Bray–Curtis distance rank boxplot comparing between-group and within-group distances. (**D**) UPGMA hierarchical clustering tree of samples based on Bray–Curtis distances.

At the OTU level, ordination based on Bray–Curtis distances showed a clear separation among stand ages (Figure 3A). The PCoA indicated that the first two axes explained 30.92% (PC1) and 23.84% (PC2) of the variation. Along PC1, one-year stands (AO) were separated from older stands (AT and AS), whereas PC2 further distinguished three-year (AT) from six-year (AS) stands. ANOSIM confirmed significant differences between groups (*R* = 0.967, *p* = 0.001). NMDS yielded consistent patterns with a low stress value (stress = 0.03; Figure 3B). A Bray–Curtis distance boxplot (Figure 3C) showed that between-group distances were higher than within-group distances, indicating pronounced separation. Within-group dispersion was lowest in AO, intermediate in AT, and slightly higher in AS. PERMANOVA further supported significant differences in community composition among stand ages (R = 0.959, P = 0.005). The UPGMA hierarchical clustering also grouped the samples by stand age (Figure 3D), supporting age-associated community turnover.

### 3.2. Community Composition and Taxonomic Differences

#### 3.2.1. Community Composition

**Figure 4:**
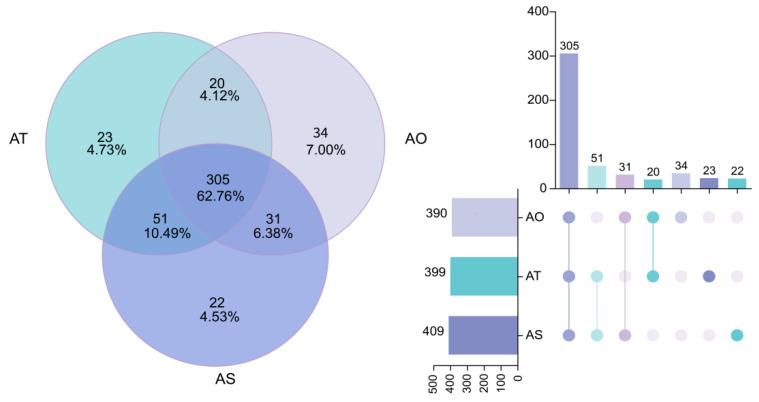
Shared and unique rhizosphere OTUs across stand ages of *H. ammodendron* (Venn diagram and UpSet plot).

The Venn and UpSet analyses (Figure 4) revealed 305 OTUs shared among all three stand ages (62.76%). The OTUs unique to AO, AT, and AS accounted for 34 (7.00%), 23 (4.73%), and 22 (4.53%), respectively. Pairwise shared OTUs were AO∩AT = 20, AO∩AS = 31, and AT∩AS = 51, indicating the presence of age-specific components beyond a stable core community.

**Figure 5:**
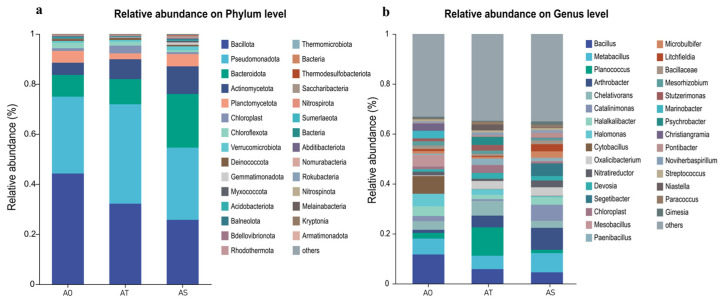
Stacked bar plots of relative abundance at the phylum (**a**) and genus (**b**) levels across *H. ammodendron* stand ages.

At the phylum level (Figure 5a), the community was dominated by *Bacillota*, *Pseudomonadota*, and *Bacteroidetes*. These groups are commonly associated with nutrient cycling and plant growth promotion in the rhizosphere and are consistent with adaptation to arid and saline conditions. At the genus level (Figure 5b), systematic differences were observed among the stand ages. Putative halotolerant and plant-beneficial genera such as *Bacillus*, *Metabacillus*, and *Planococcus* (*Bacillota*); drought-adapted decomposers such as *Arthrobacter* (*Actinomycetota*); and *Halophiles* such as *Halomonas* (*Pseudomonadota*) displayed age-dependent abundance patterns. PERMANOVA (R = 0.959, P = 0.005) further supported the significant compositional differences among stand ages.

**Figure 6:**
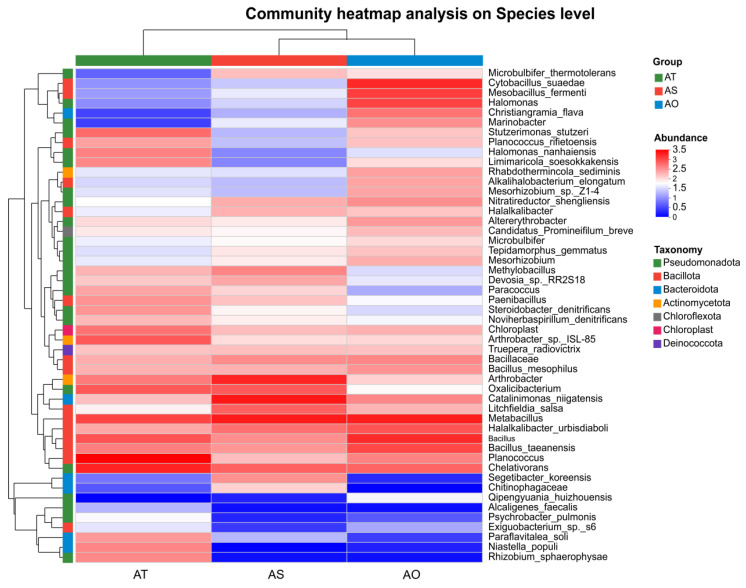
Heatmap of species-level relative abundance (z-score standardized by species).

At the species level (Figure 6), samples clustered clearly by stand age, and two opposing species clusters were apparent. Cluster I was more abundant in AT and AS, and included species such as *Segetibacter koreensis*, *Litchfieldia salsa*, *Ramlibacter montanisoli*, and *Pontibacter flavimaris*, which are often linked to polysaccharide/complex substrate degradation and stress tolerance. Cluster II was relatively high in AO and included *Halomonas* sp., *Neobacillus* sp., and *Mesorhizobium* sp., which are salt-tolerant early colonizers. Overall, older stands tended to accumulate degraders and stress-resistant functional groups.

**Figure 7:**
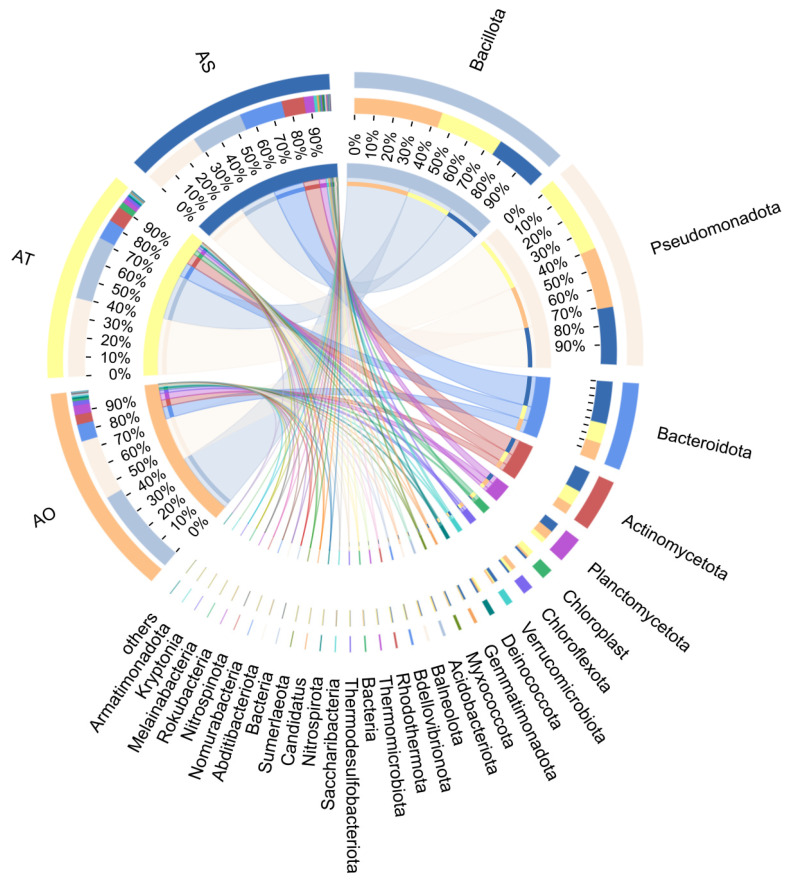
Chord diagram showing relative abundance contributions between stand ages and major bacterial phyla.

A chord diagram (Figure 7) further illustrates the relative contributions of stand age groups to the major bacterial phyla. All groups were dominated by *Bacillota* and *Pseudomonadota*, with thicker ribbons in the AS indicating increased contributions, whereas *Bacteroidetes* contributed relatively more to AO. Overall, the phylum-level framework was relatively stable, while clear age-associated shifts and replacements occurred at the genus and species levels.

#### 3.2.2. Differential Taxa

**Figure 8:**
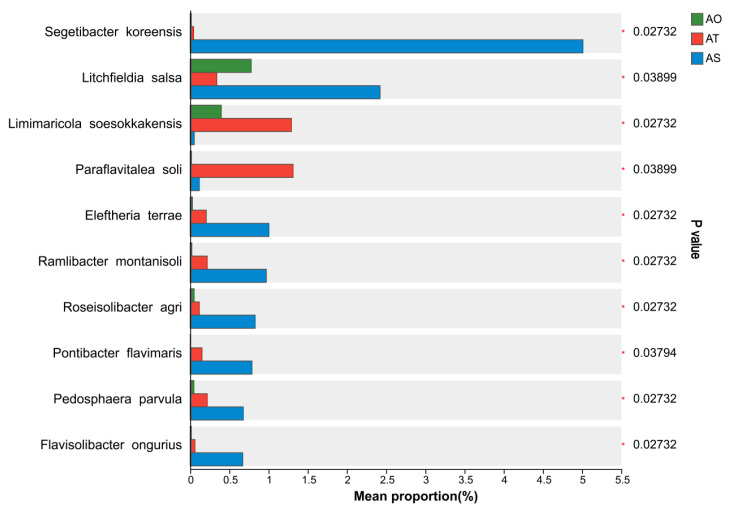
Differential test analysis of species among stand ages.

Multigroup comparisons at the species level identified ten species with significant differences among stand ages (P = 0.027, 0.039, 0.027, 0.039, 0.027, 0.027, 0.027, 0.038, 0.027, 0.027 < 0.05; Figure 8). Most of these species were significantly enriched in the 6-year rhizosphere (AS), including *Segetibacter koreensis*, *Litchfieldia salsa*, *Eleftheria terrae*, *Ramlibacter montanisoli*, *Roseisolibacter agri*, *Pontibacter flavimaris*, *Pedosphaera parvula*, and *Flavisolibacter longurius*, generally following an “AS > AT ≈ AO” pattern. In contrast, *Limimaricola soesokkakensis* and *Paraflavitalea soli* showed the highest relative abundances in the three-year rhizosphere (AT). These taxa are often associated with polysaccharide and complex organic matter degradation and may exhibit halotolerance and plant-beneficial potential, consistent with the observed age-associated shift toward degrader and stress-tolerant functional groups in older stands.

**Figure 9:**
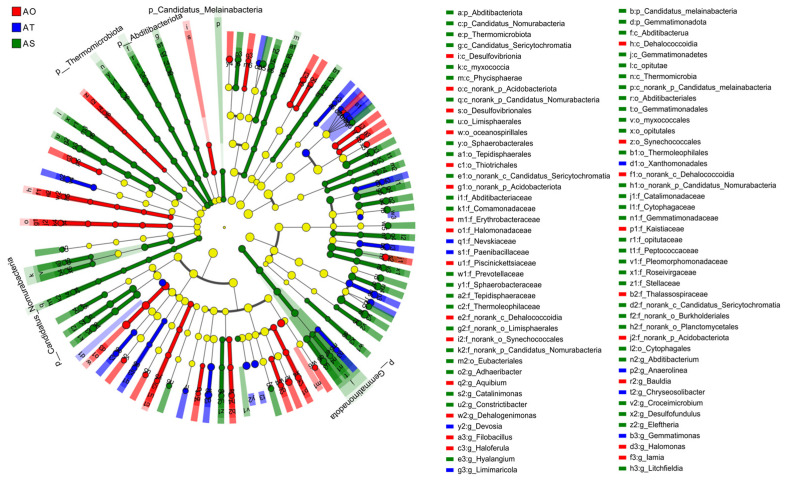
LEfSe multi-level discriminant analysis of differentially abundant lineages across stand ages.

The LEfSe cladogram results (Figure 9) indicate pronounced phylogenetic differentiation among the three groups. Overall, the six-year group (AS) contained the largest number of enriched lineages, followed by the one-year group (AO), whereas the three-year group (AT) had fewer discriminant lineages. Indicator taxa in the AO included *Altererythrobacter*, *Bacillus*, and *Halomonas*, which are typical early colonizers or stress-tolerant taxa in saline and arid environments. AT is characterized by lineages, such as *Paenibacillaceae*, *Devosia*, *Rhizobium*, *Limimaricola*, and *Xanthomonadales*, suggesting an intermediate assembly stage emphasizing plant growth promotion, nitrogen cycling, and organic matter metabolism. AS is enriched in taxa such as *Segetibacter*, *Ramlibacter*, *Roseisolibacter*, *Pontibacter*, and *Flavisolibacter*, many of which are associated with the degradation of complex organic matter and adaptation to arid-saline conditions. Collectively, LEfSe recapitulated a succession pattern from stress-tolerant colonizers (AO) to putative plant-beneficial and nitrogen-cycling taxa (AT) and to complex substrate degraders with enhanced stress tolerance (AS).

### 3.3. Community Interactions and Predicted Functional Profiles

#### 3.3.1. Co-Occurrence Network

**Figure 10:**
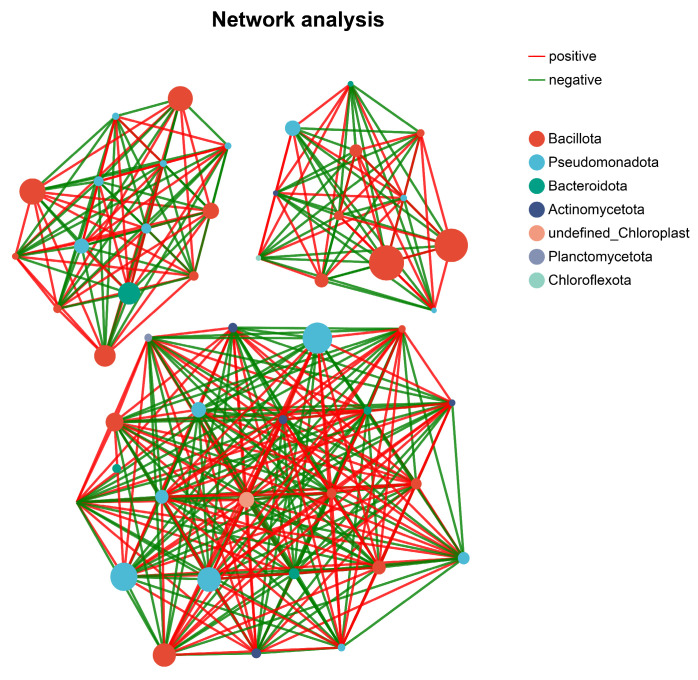
Co-occurrence network of rhizosphere bacterial OTUs across samples.

A co-occurrence network based on Spearman’s correlations at the OTU level revealed a highly connected, multi-modular structure (Figure 10). Positive correlations greatly outnumbered negative correlations, suggesting that potential co-occurrence and coexistence relationships dominated antagonistic interactions. At the phylum level, *Bacillota* and *Pseudomonadota* contained many hub nodes and formed dense subnetworks with *Bacteroidetes* and *Actinomycetota*, indicating potential interactions among taxa involved in organic matter degradation, nutrient transformation, and stress adaptation. These highly connected nodes overlapped with the candidate functional taxa identified above, suggesting that they represent structurally central taxa within the inferred association network.

#### 3.3.2. Predicted Functional Profiles

**Figure 11:**
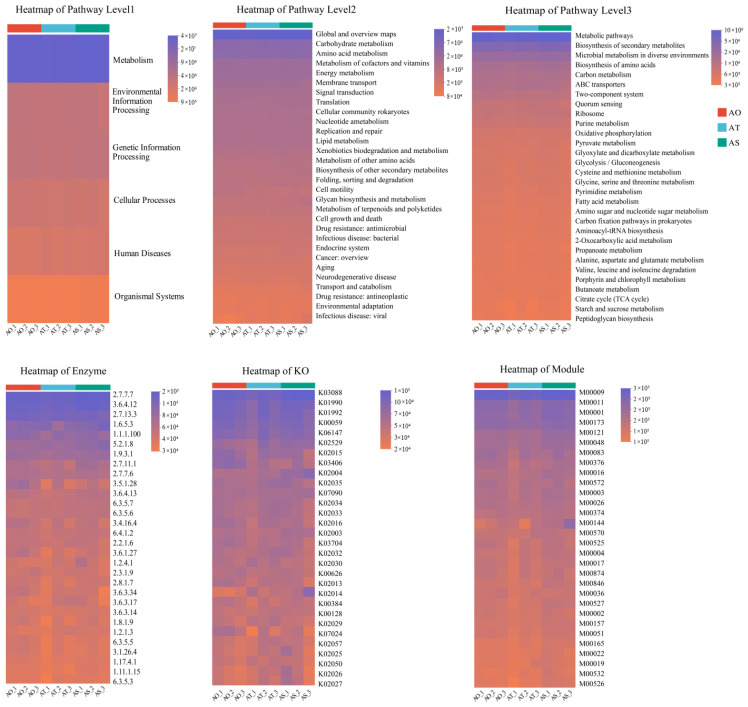
Predicted KEGG functions (Levels 1–3) and heatmaps of Enzyme (EC), KO, and KEGG Module profiles.

The PICRUSt2 inference suggested that functional profiles were broadly conserved across stand ages (Figure 11). At KEGG Level 1, metabolism accounted for the largest proportion of predicted functions, followed by Environmental Information Processing, Genetic Information Processing, and Cellular Processes, whereas pathways related to Human Diseases and Organismal Systems were minor. At KEGG Levels 2 and 3, the major pathways included carbohydrate metabolism, amino acid metabolism, metabolism of cofactors and vitamins, energy metabolism, membrane transport (e.g., ABC transporters), two-component systems, quorum sensing, core carbon metabolism (e.g., glycolysis and the TCA cycle), fatty acid metabolism, and starch and sucrose metabolism. Heat maps of the EC, KO, and KEGG modules showed a shared core enzymatic and functional gene repertoire dominated by carbon degradation, energy acquisition, and amino acid metabolism. Several metabolism- and transport-related features were slightly higher in AT and AS than in AO, suggesting a gradual increase in potential organic substrate turnover and environmental responses in older stands, whereas the overall functional backbone remained relatively stable.

**Figure 12:**
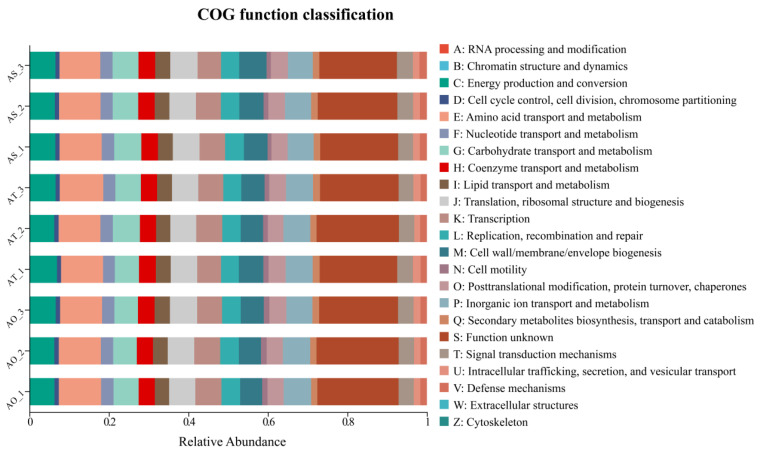
COG functional classification of predicted bacterial gene families.

MetaCyc predictions were largely consistent with KEGG, with amino acid biosynthesis and degradation, fatty acid biosynthesis and oxidation, the TCA cycle, and branched-chain amino acid biosynthesis among the dominant pathways and slightly higher predicted abundance in AT and AS. COG functional categories (Figure 12) similarly indicated the dominance of energy production and conversion, amino acid and carbohydrate transport and metabolism, transcription and translation, cell wall and membrane biogenesis, and inorganic ion transport and metabolism; modest increases in energy metabolism, secondary metabolite biosynthesis, signal transduction, and defense mechanisms were observed in AT and AS. Overall, the functional prediction suggests a transition from basic maintenance to more efficient resource utilization and environmental responsiveness with stand age, which could provide a functional context relevant to host performance.

#### 3.3.3. Stand-Age Succession of Indicator and Candidate Functional Groups

The phylogenetic discrimination by LEfSe and the multi-group comparisons together support a stage-wise turnover of indicator taxa: AO was characterized by salt-tolerant early colonizers (e.g., *Altererythrobacter*, *Bacillus*, and *Halomonas*); AT was enriched in putative plant-beneficial and nitrogen-cycling taxa (e.g., *Paenibacillaceae*, *Devosia*, *Rhizobium*, *Limimaricola*, and *Xanthomonadales*), suggesting enhanced plant growth promotion and organic matter metabolism; AS was dominated by *Chitinophagaceae–Cytophagales* lineages and related genera (e.g., *Segetibacter* and *Flavisolibacter*) linked to polysaccharide/recalcitrant organic matter degradation and stress tolerance. Accordingly, we grouped representative taxa into three candidate functional guilds: (i) AO, halotolerance and early colonization; (ii) AT, plant growth promotion and nitrogen cycling with enhanced organic metabolism; and (iii) AS-complex-substrate degradation and stress resistance. Within each group, these guilds exhibited a replacement trend from AO to AT to AS, consistent with the opposing gradients observed in the species heatmap and the increased contributions of the dominant phyla in AS. Together with the network and functional prediction results, these findings suggest an ordered functional succession from a colonizer-dominated rhizosphere (AO) to a growth-promotion/nitrogen-cycling stage (AT) to a degrader and stress-resistance stage (AS).

### 3.4. Soil Physicochemical Properties

To evaluate whether microbial differences could be attributed to soil physicochemical variations, we compared pH, SOM, TN, TP, TK, AN, AP, and AK among stands of different ages. No significant differences were detected among groups (Kruskal–Wallis test; Benjamini–Hochberg adjusted *q* ≥ 0.10; Table 3), and soil PCA did not show clustering by stand age. Therefore, the observed microbial community separation is unlikely to be explained solely by the measured soil physicochemical differences, which should be interpreted as a stand age–associated pattern under broadly comparable measured soil backgrounds, rather than as evidence of a fully partitioned stand-age effect.

## 4. Discussion

### 4.1. Stand Age as an Ecological Filter Shaping Rhizosphere Microbial Assembly

In broadly comparable soil physicochemical backgrounds, alpha diversity remained relatively stable among stand ages, whereas beta diversity and overall community composition exhibited pronounced age-associated turnover. This pattern suggests that stand age–associated differences in this system are primarily expressed through reorganization of community structure rather than simple richness changes. Unlike previous studies that predominantly attributed rhizosphere microbiome variation to abiotic gradients, our findings highlight plant stand age as an integrative ecological dimension associated with microbial succession. As *H. ammodendron* stands develop, progressive shifts in root exudation, litter input, and belowground resource allocation may contribute to coordinated restructuring of rhizosphere bacterial communities. These patterns are compatible with plant-mediated ecological filtering, which interacts with environmental constraints to structure microbial communities over time.

### 4.2. Taxonomic Turnover Within a Conserved Higher-Taxonomic Framework

Although the dominant phyla, including *Bacillota*, *Pseudomonadota*, and *Bacteroidetes*, formed a relatively stable backbone across stand ages, genus- and species-level composition changed systematically along the age gradient. Younger stands were enriched in stress-tolerant and early colonizing taxa, which is consistent with communities adapted to more unstable rhizosphere environments. Intermediate stands showed increased representation of putative plant-associated and nitrogen-cycling bacteria, potentially reflecting strengthened plant–microbe interactions during active growth phases. In older stands, enrichment of taxa linked to complex carbon turnover and stress adaptation suggests a shift toward microbial communities capable of utilizing more recalcitrant substrates. Rather than indicating abrupt community replacement, these results imply gradual transitions in microbial ecological strategies accompanying shrub development.

### 4.3. Network Organization and Predicted Functional Differentiation

Co-occurrence network analysis revealed modular organization across stand ages, indicating structured patterns of microbial associations within the rhizosphere. Although correlation-based networks inferred from relative abundance data represent putative associations rather than direct ecological interactions, the observed modularity suggests that nonrandom community organization is potentially related to niche differentiation. Functional inference based on PICRUSt2 indicated that a conserved core metabolic repertoire was shared among stand ages, whereas predicted ecological functions exhibited gradual differentiation along the age gradient. Because PICRUSt2 infers functional potential from 16S rRNA gene data, these predictions represent putative metabolic capacities rather than directly measured activities and should therefore be interpreted with caution. These findings are consistent with the notion that rhizosphere functional profiles may shift through changes in the relative weighting of ecological functions within a stable higher taxonomic framework, rather than through wholesale functional replacement [22,43].

### 4.4. Ecological Implications for Desert Shrub Systems

The stand age-associated succession observed in the present study provides insights into plant-mediated microbiome development in arid ecosystems. Long-lived desert shrubs, such as *H. ammodendron*, progressively modify their rhizospheres through cumulative biological inputs, potentially generating temporally structured microbial habitats. From an ecological perspective, these results suggest that microbial community assembly in extreme environments may reflect interactions between deterministic plant-associated filtering and environmental constraints, providing a community-level perspective on how long-lived desert shrubs shape rhizosphere microbial assembly over time.

In the context of parasitic plant systems, previous studies have shown that microbiomes of parasitic plants can resemble those of their hosts, and that rhizosphere similarity may influence parasitism dynamics. Although parasitism-related phenotypes were not measured in this study, the observed age-associated shifts in predicted nitrogen cycling, substrate utilization, and stress response potentials provide a bacterial perspective that may be relevant to host suitability. However, these implications remain inferential and warrant further experimental validation.

### 4.5. Study Limitations and Future Directions

This study had several limitations. First, the relatively limited number of biological replicates may constrain statistical power and reduce the generalizability of the observed patterns. Therefore, the stand age–associated differences observed here should be interpreted cautiously and validated in future studies with larger sample sizes and broader spatial replication. Second, functional profiles were inferred from 16S rRNA gene data and therefore represented predicted rather than directly measured metabolic capacities. Third, although soil physicochemical properties were broadly comparable among the sites, additional multivariate analyses, such as Mantel tests, RDA/CCA, or variation partitioning, were not performed; therefore, the relative contribution of stand age versus subtle environmental variation should be interpreted cautiously. Fourth, temporal inference was based on space-for-time substitution using stand age rather than longitudinal monitoring. Future work integrating larger-scale sampling, metagenomics, fungal and archaeal profiling, and experimental manipulation will be necessary to validate the observed patterns and disentangle deterministic and stochastic processes underlying plant-associated microbial assembly in desert ecosystems.

## 5. Conclusions

Under broadly similar soil physicochemical conditions (pH, SOM, TN, TP, AN, AP, AK, and EC), rhizosphere bacterial communities associated with one-, three-, and six-year-old *H. ammodendron* stands showed no significant differences in alpha diversity but exhibited clear stand age separation in beta diversity and community structure, indicating a pronounced stand age–associated pattern. *Bacillota*, *Pseudomonadota*, and *Bacteroidetes* constituted a stable phylum-level backbone, whereas genus and species composition and inferred functions were consistent with an ordered succession from a halotolerant colonizer stage (one-year), to a stage enriched in putative plant growth-promotion and nitrogen cycling (three-year), and to a stage characterized by enhanced polysaccharide and recalcitrant organic matter degradation and stress resistance (six-year). The co-occurrence network was highly connected and modular, with predominantly positive associations, and *Bacillota* and *Pseudomonadota* contained many hub nodes. PICRUSt2 predicted a conserved core metabolic repertoire across stand ages, with gradual increases in pathways related to carbon degradation, energy metabolism, membrane transport, and environmental responses in older stands. Overall, these findings suggest a progressive age-associated shift in predicted functional profiles in the rhizosphere bacteriome of *H. ammodendron* and generate testable hypotheses regarding plant–microbiome interactions in parasitic systems.

## Figures and Tables

**Table 1:** Methods used for soil physicochemical measurements.

Variable	Method
pH	Potentiometric method
TN	Kjeldahl method
TP	NaOH alkaline fusion–molybdenum antimony colorimetry
TK	NaOH fusion method
AN	Alkaline hydrolysis diffusion method
AP	Spectrophotometry
AK	Ammonium acetate extraction
SOM	Potassium dichromate oxidation titration

**Table 2:** Alpha diversity indices of rhizosphere bacterial communities from one-, three-, and six-year-old *H. ammodendron* stands.

Group	Diversity Index		Richness Index			Uniformity Index
	Simpson	Shannon	Sobs	Ace	Chao	Coverage
AO1	0.0245	4.5867	556	645.3062	642.25	0.9933
AO2	0.0413	4.3602	599	738.5332	735.07	0.9909
AO3	0.0154	4.9944	637	728.8676	734.44	0.9929
AT1	0.0292	4.5146	545	592.7240	615.58	0.9950
AT2	0.0470	4.2341	513	635.1320	670.55	0.9920
AT3	0.0283	4.7629	606	688.5703	699.94	0.9933
AS1	0.0475	4.3270	561	649.4628	664.48	0.9930
AS2	0.0356	4.6194	616	701.0511	711.86	0.9929
AS3	0.0380	4.5432	646	736.0756	739.43	0.9926

**Table 3:** Soil physicochemical properties of rhizosphere soils across stand ages.

Stand Age	pH	TN (g/kg)	TP (g/kg)	TK (g/kg)	AN (mg/kg)	AP (mg/kg)	AK (mg/kg)	SOM (g/kg)
AO	8.83	0.129	0.591	6.76	11.672	7.171	159.783	2.660
AT	8.35	0.165	0.604	10.69	9.114	7.381	377.900	3.186
AS	8.82	0.101	0.649	7.08	10.697	8.086	185.962	2.547

## Data Availability

The data presented in this study are available on request from the corresponding author.

## Abbreviations

The following abbreviations are used in this manuscript:

AO	1-year-old *Haloxylon ammodendron*
AT	3-year-old *Haloxylon ammodendron*
AS	6-year-old *Haloxylon ammodendron*
